# Clinical Evaluation of the Torq Zero Delay Centrifuge System for Decentralized Blood Collection and Stabilization

**DOI:** 10.3390/diagnostics11061019

**Published:** 2021-06-02

**Authors:** Kyungjin Hong, Gabriella Iacovetti, Ali Rahimian, Sean Hong, Jon Epperson, Clara Neal, Tifany Pan, Angela Le, Eric Kendall, Kory Melton, Greg J. Sommer, Ulrich Y. Schaff

**Affiliations:** Sandstone Diagnostics, Inc., 6624 Owens Drive, Pleasanton, CA 94588, USA; khong@sandstonedx.com (K.H.); giacovetti@sandstonedx.com (G.I.); rahimian.ali@aol.com (A.R.); sean.hs.hong@gmail.com (S.H.); jepperson@sandstonedx.com (J.E.); plumberry00@gmail.com (C.N.); tpan@sandstonedx.com (T.P.); angela97le@ucla.edu (A.L.); eric.kendall@gmail.com (E.K.); kmelton31596@gmail.com (K.M.); gsommer@sandstonedx.com (G.J.S.)

**Keywords:** plasma separation tube (PST), portable centrifuge, point-of-collection, blood sample preparation, blood sample stabilization, blood collection device

## Abstract

Blood sample collection and rapid separation—critical preanalytical steps in clinical chemistry—can be challenging in decentralized collection settings. To address this gap, the Torq™ zero delay centrifuge system includes a lightweight, hand-portable centrifuge (ZDrive™) and a disc-shaped blood collection device (ZDisc™) enabling immediate sample centrifugation at the point of collection. Here, we report results from clinical validation studies comparing performance of the Torq System with a conventional plasma separation tube (PST). Blood specimens from 134 subjects were collected and processed across three independent sites to compare ZDisc and PST performance in the assessment of 14 analytes (K, Na, Cl, Ca, BUN, creatinine, AST, ALT, ALP, total bilirubin, albumin, total protein, cholesterol, and triglycerides). A 31-subject precision study was performed to evaluate reproducibility of plasma test results from ZDiscs, and plasma quality was assessed by measuring hemolysis and blood cells from 10 subject specimens. The ZDisc successfully collected and processed samples from 134 subjects. ZDisc results agreed with reference PSTs for all 14 analytes with mean % biases well below clinically significant levels. Results were reproducible across different operators and ZDisc production lots, and plasma blood cell counts and hemolysis levels fell well below clinical acceptance thresholds. ZDiscs produce plasma samples equivalent to reference PSTs. Results support the suitability of the Torq System for remotely collecting and processing blood samples in decentralized settings.

## 1. Introduction

The majority of clinical chemistry assays are conducted on plasma or serum prepared by centrifuging whole blood in plasma separation tubes (PSTs) or serum separation tubes (SSTs). Sample centrifugation most often occurs in centralized laboratories on the frontend of analytical workflows. Most clinical guidelines recommend spinning PSTs within 2 h of collection, and SSTs within 1 h of collection following a 30 min clotting step, although earlier separation is recommended when possible to preserve the in vivo state of the sample [1]. Delays between blood sample collection and centrifugation lead to sample hemolysis, cell degradation, and overall diminished sample quality which can negatively impact analytical sensitivity, accuracy, and reproducibility [2,3,4,5]. Indeed, preanalytical errors account for up to 75% of all diagnostic errors, of which sample hemolysis is the most common source of preanalytical errors, accounting for an estimated 60% of rejected samples [6].

Implementing conventional centrifugation procedures with swing-out or swinging bucket centrifuges in decentralized blood collection settings is challenging due to the size, power requirements, cycle time, and operating procedures for conventional laboratory centrifuges. Alternatively, portable centrifuges capable of processing conventional venipuncture blood tubes tend to be bulky, power hungry, limited to fixed angle configurations, and troublesome to maintain and balance and have therefore been only marginally useful for mobile phlebotomy applications. As a result, blood tubes collected by mobile phlebotomists are typically transported to laboratories unseparated, and decentralized access to lab testing for sensitive analytes remains limited [5].

Many efforts have been made to develop portable or microfluidic-based plasma separation devices as a replacement for centrifuges [7], including a paper-based centrifuge developed by Prakash lab [8]. Other microdevices for plasma separation utilize various passive separation techniques including sedimentation [9], filtration [10], lateral displacement [11], and hydrodynamic effects [12]. Of these, only filtration-based approaches have been commercialized, such as Whatman Filters. Most microdevices work only with small volume samples (i.e., capillary blood volumes much less than 1 mL), work with a limited range of hematocrit, may require blood sample dilution prior to separation, and may require costly pumping mechanisms. Such limitations impede the microdevices for plasma separation being translated into commercial devices for point-of-care blood collection and separation.

**Figure 1:**
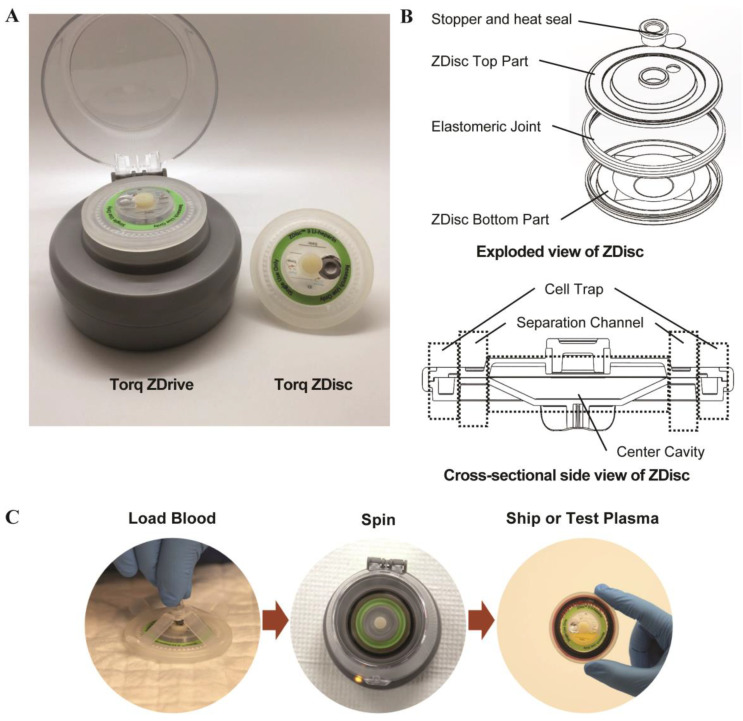
(**A**) Torq Zero Delay Centrifugation System. (**B**) Schematic representation of Torq ZDisc showing main components (Top; exploded view) and key structural features (Bottom; cross-sectional side view). (**C**) Three-step workflow of ZDisc: load, spin, and preparation for shipping or testing of plasma.

To address this gap in decentralized blood specimen collection and stabilization, we have developed the Torq™ Zero Delay Centrifugation System (Figure 1). The Torq system comprises the Torq ZDisc™ (“ZDisc”)—a disc-shaped blood container designed for vacuum-facilitated collection and centrifugal plasma separation of 3.5 mL venous whole blood—and the Torq ZDrive™—a compact (4-inch diameter, < 1 lb), portable, battery-powered, easy to use, and low-cost centrifuge designed to spin the ZDisc. The Torq ZDisc is an injection molded, mesofluidic device with an aerodynamic structure that combines the centrifugation rotor with the collection vessel to optimize the speed, power input, and footprint required for sample separations. The ZDisc’s smooth interior pathway efficiently facilitates blood separation without the use of separator gels in order to minimize blood cell rupture and chemical interference, therefore enabling faster whole blood separation compared to conventional blood collection tubes while avoiding the potential interferences caused by gel contact [13,14,15]. As shown in Figure 1B, the ZDisc consists of three compartments: (1) center cavity where whole blood sample is collected (internal volume ~4 mL); (2) cell trap where blood cells are retained after separation (volume ~1.8 mL); and 3) separation channel between them, which opens upon the centrifugal force (default volume ~0.2 mL and expands up to ~1 mL). The ZDisc’s compact design enables g-forces that meet or exceed conventional centrifuges (median: 2920 g, 95% CI: 2670 g–3160 g), while minimizing the specimen’s radial separation distance, resulting in rapid (5 min) separation with low energy input (2 AA lithium batteries for at least five complete runs). The Torq system allows healthcare professionals to easily collect whole blood through routine venipuncture and immediately separate and stabilize plasma samples at the point of collection.

Here, we describe the findings from blinded, cross-sectional multisite clinical studies conducted to evaluate the performance of the ZDisc coated with lithium heparin as an anticoagulant in comparison with a reference plasma separation tube when measuring electrolytes potassium (K), sodium (Na), chloride (Cl), and calcium (Ca); kidney function analytes blood urea nitrogen (BUN) and creatinine; liver function parameters aspartate transaminase (AST), alanine aminotransferase (ALT), alkaline phosphatase (ALP), total bilirubin (T.Bili), albumin (Alb), and total protein (TP); and lipids including cholesterol (Chol) and triglycerides (TRIG)—most of which require blood separation within a limited time frame for valid results [16].

## 2. Materials and Methods

### 2.1. Chemicals and Reagents

Lithium heparin (CAS no. 9045-22-1; Sigma-Aldrich, St. Louis, MO, USA) was spray-coated on the ZDiscs as an anticoagulant. ISE Diluent Generation II (Roche Diagnostics, Mannheim, Germany) was used for modifying a small number of samples with decreased analyte concentrations. Potassium chloride enriched lactated Ringer’s solution was prepared using Ringer’s lactate solution (Hi-Media Laboratories Private Limited, Mumbai, India) and potassium chloride (CAS no. 7447-40-7; VWR Chemicals, Solon, OH, USA) and then used in modifying a small number of samples to achieve higher analyte concentrations. In order to extend the analytical range, exogenous ALT (BioVision Inc., Mountain View, CA, USA) and ALP (Roche Diagnostics, Mannheim, Germany) were added to the potassium enriched lactated ringer’s solution for 2 separate samples.

### 2.2. Subject Recruitment

The study was a cross-sectional, multi-site investigation conducted at three sites within the United States (ClinicalTrials.gov study protocol identifier: NCT04206839). Blood samples were collected from subjects under Salus Institutional Review Boards (IRB) #00006833, #00006834, and #00009473 and Indiana University IRB #00000219. All subjects who participated in the study gave informed consent. All patient-facing material was also subject to IRB review. The principles of good clinical practice (GCP) and reporting of adverse events were followed. Eligible subjects were at least 18 years of age, at least 110 pounds, and not pregnant. All subjects were screened using AABB (formerly American Association of Blood Banks) screening questionnaires. Efforts were made to recruit otherwise healthy subjects with conditions that could result in metabolic or electrolyte imbalance, but no specific metrics for health status were recorded. Demographic subject information was collected for a descriptive summary.

### 2.3. Performance Comparison Study

One hundred thirty-four (134) patient samples across 3 sites were used to compare the performance of the ZDisc with the BD Vacutainer^®^ Plus PST II with lithium heparin (Becton Dickinson, Franklin Lakes, NJ, USA), guided by the recommendations contained in the CLSI GP34-AE and GP44-A4 guidelines [1,17]. Although a larger subject population would be required for the validation of a specimen container for use with all the possible blood plasma analytes, for the current study, the recommendations associated with CLSI GP34-AE were followed (a minimum sample size of *n* = 40 at each of 3 sites, a minimum total of 120 subjects). Further validation will be necessary to establish method validity with analytes outside of the 14 tested in this study.

A ZDisc and a PST were drawn from each subject in a randomized order. From each donor, 3.5 mL and 4 mL of venous whole blood were collected into a ZDisc and a PST, respectively. ZDiscs were spun using the Torq ZDrive, and PSTs were spun following the manufacturer’s recommended spin settings of 10 min at 1200 g using conventional swing-out centrifuges (BLC-40D, Aiken Corp; Heraeus Megafuge 1.0R 3062, Baxter; Eppendorf^®^ 5702R, Sigma Aldrich). Each pair of a ZDisc and a PST were processed by one operator, a trained healthcare professional (HCP). Plasma from each ZDisc and each PST was aliquoted into two coded transport tubes and kept frozen at −20 °C until testing. Analyte concentrations in plasma from both ZDisc and PST were measured using two different chemistry analyzers—a Beckman Coulter AU 5820 analyzer and Roche Cobas C-501 analyzer, at two central laboratories in parallel. Note that lipid concentrations were assessed using only one chemistry analyzer (Roche Cobas C-501 analyzer) due to differences in the panel configuration between the two analyzers. Analyzer operators were blinded to the identity of the blood container used to collect and process each sample. Each analyzer used in the performance comparison study was operated by an independent laboratory in which daily controls were run for each analyte on reference samples according to recommended practices of the College of American Pathologists (CAP). All measurement processes followed good laboratory practices (GLP), including traceable reagent lots and training records.

A set of primary medical decision levels (MDLs) [18,19,20,21,22,23] was selected for each analyte to assess coverage of each analyte’s reference interval (see Table 1). Consensus MDLs were defined by the upper end of the reference interval and were used for the analysis herein. In addition to the MDLs listed in Table 1, select secondary or condition-specific MDLs at more extreme intervals were assessed for the estimated mean % bias for some analytes (see Supplementary Table S2 for more information).

In order to augment the range of analyte concentrations, including both extreme high and low levels of select analytes for which native samples in this range were not available (K, Na, Cl, AST, and ALP), blood samples from six subjects were modified as follows. Blood from an individual subject was collected into conventional blood collection tubes, which were metered into one 50 mL conical tube, and slightly diluted with a solution to increase or decrease the concentration of the select analytes (K, Na, Cl, AST, and ALP) as indicated. Samples from one subject were diluted 18% with a potassium enriched lactated ringer’s solution to raise the concentration of K, Na, and Cl. Samples from two subjects were diluted 13% and 15% with a K, AST, and ALP enriched lactated Ringer’s solution to increase the concentration of K, Na, Cl, AST, and ALP. Samples from three subjects were diluted with an ISE calibration solution utilized to dilute the concentrations of K, Na, and Cl by 10%, 14%, and 19%, respectively. After dilution, the pooled sample was drawn into ZDiscs and PSTs in a randomized draw order. For a detailed description of the sample modification method, please see the Supplementary Material, including Figure S1. The sample modification method was found to provide valid analytical results as demonstrated by an *n* = 10 sample modification method validation study, whose results are also provided in the Supplementary Material (Table S25).

### 2.4. Precision Study

Thirty-one (31) patient samples across 3 sites were used for the evaluation of precision, based on CLSI EP-5-A3 guidelines [24]. Each subject had blood drawn into 5 ZDiscs from three different lots, including 3 ZDiscs from one lot (i.e., Lot A) and 2 ZDiscs from another two different lots (i.e., Lot B and Lot C), in a randomized draw order by trained healthcare professionals. To assess lot-to-lot precision, 3 different lots of ZDiscs from each subject were processed by the same operator using the Torq ZDrive. To assess inter-operator precision, 3 ZDiscs from a single lot (i.e., Lot A) were processed using ZDrives by 3 different operators. The first ZDisc from Lot A for each subject was used to evaluate both lot-to-lot precision and inter-operator precision. In addition, for each subject, the operator who processed a set of ZDiscs for lot-to-lot precision also participated in the inter-operator precision study as one of the three test operators.

In order to augment the range of analyte concentrations, select samples from individual subjects were modified similarly to the performance comparison study. Briefly, blood from an individual subject was collected into conventional blood collection tubes, metered into one 50 mL conical tube, and then slightly diluted with a solution to increase or decrease the concentration of select analytes (K, Na, Cl, AST, and ALP). Specifically, samples from two subjects at each of the three sites were diluted 18% with a potassium enriched lactated ringer’s solution, and samples from two subjects at each of the three sites were diluted 19% with an ISE calibration solution. After dilution, the pooled sample from each subject was drawn into 5 ZDiscs per sample in a randomized draw order. The method of modifying samples was validated prior to the clinical trial.

To assess lot-to-lot precision, 3 different lots of ZDiscs from each subject were processed by the same operator using the Torq ZDrive. To assess inter-operator precision, 3 ZDiscs from a single lot were processed using ZDrives by 3 different operators. The first ZDisc from Lot A for each subject was used to evaluate both lot-to-lot precision and inter-operator precision. In addition, for each subject, the operator who processed a set of ZDiscs for lot-to-lot precision also participated in the inter-operator precision study as one of the three test operators.

Plasma samples separated using ZDiscs were transferred into 2 coded transport tubes and kept frozen until testing at a central laboratory. Measurements of all analyte concentrations were completed on a Roche Cobas C-501 analyzer by laboratory personnel who were blinded to the identity of the tubes. Intra- and inter-variability for lot-to-lot precision and inter-operator precision studies were calculated using ANOVA.

### 2.5. Plasma Quality

Plasma quality was assessed by measuring hemolysis (i.e., concentration of free hemoglobin), red blood cell (RBC), white blood cell (WBC), and platelet (PLT) count in 10 subject specimens separated using ZDiscs from three different production lots.

Plasma-free hemoglobin was assessed by calibrated spectrophotometry by a Beckman DU530 spectrophotometer using the 415 nm, 450 nm, and 700 nm wavelengths [25]. A plasma hemoglobin level of 100 mg/dL or higher significantly interferes with many assays and may be used as a rejection threshold, above which blood collection may need to be repeated [26,27]. Similarly, the acceptable limits for cell contamination for the separated plasma were defined as 6 × 10^6^/mL, 0.1 × 10^6^/mL, and 50 × 10^6^/mL for RBCs, WBCs, and PLTs, respectively [28].

### 2.6. Data Analysis

All data analysis was conducted on RStudio Desktop 1.1.463 (The R Foundation), including variance component analysis, Deming regression analysis, and Bland–Altman plot generation. In particular, ‘VCA package’ v 1.1.1 was employed for the ANOVA-type estimation of variance components for linear mixed models. In addition, ‘mcrpackage’ v 1.2.1 was used to implement Deming regression analysis and to estimate the bias, 95% CI for the bias at MDLs, and the overall mean % bias. All statistical tests were 2-tailed and performed at the 5% significance level, unless stated otherwise.

## 3. Results

### 3.1. Performance Comparison

Subjects included in the performance comparison study were 50.76 years old on average (range 18–79), with 20.2% male and 78.4% female (note: two subjects refused to answer). See Supplementary Table S1 for subject demographic details.

**Figure 2:**
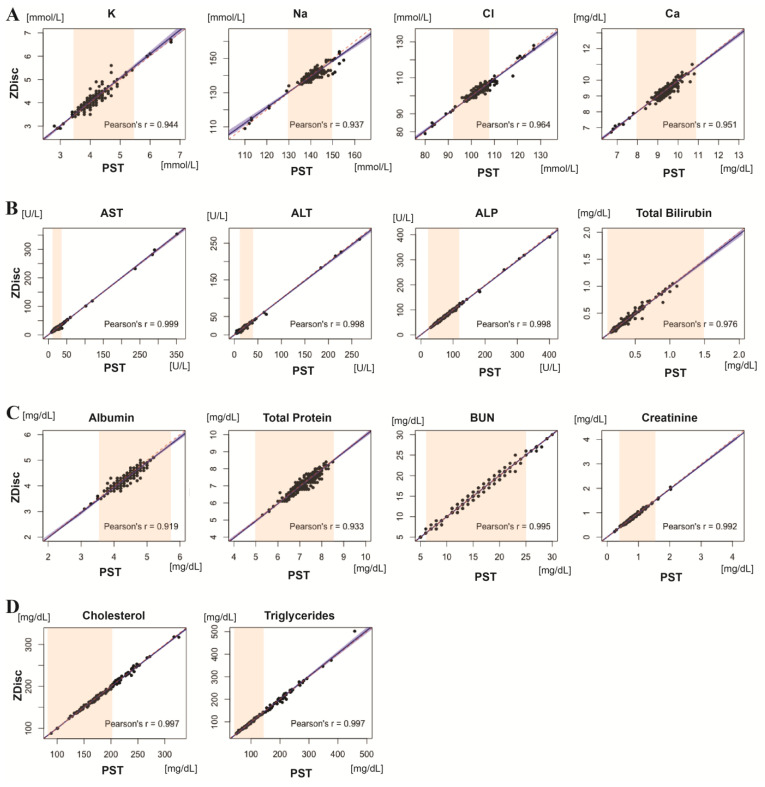

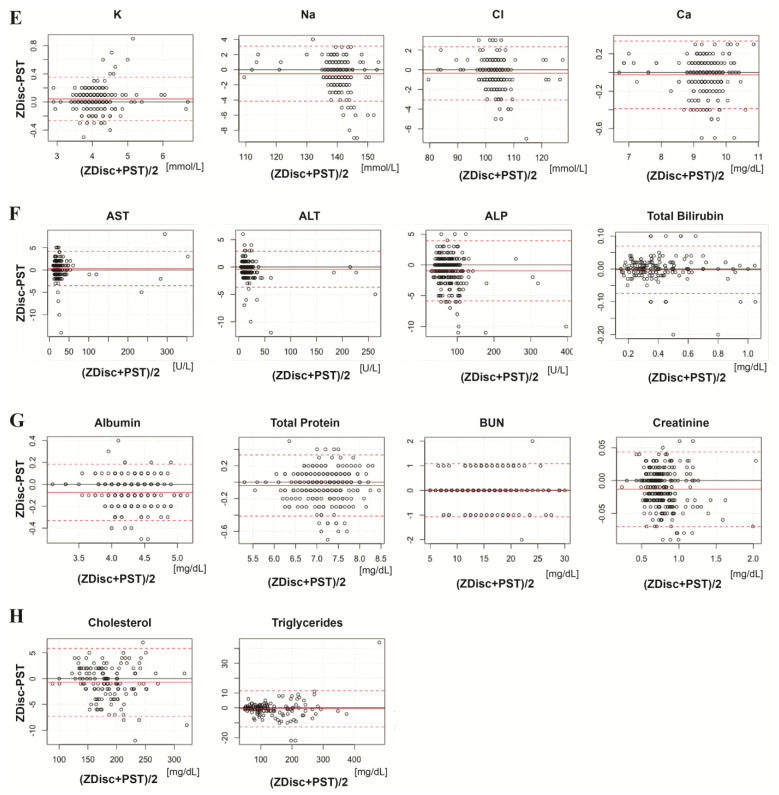
Scatter plots representing all 14 analytes results for ZDisc vs. vacutainer PST with Deming regression line with 95% CI (blue), reference interval for each analyte (orange), and identity line (red dots) across all sites and analyzers (**A**–**D**); Bland–Altman plots with mean (red solid line) and +/−2*SD (red dotted line) (**E**–**H**).

Summaries of Deming regression analysis and the bias between ZDiscs and PSTs across all sites and across all analyzers are shown in Figure 2. For all 14 analytes, Pearson’s coefficient (r) was close to 1 (r > 0.9), and the 95% confidence intervals of the slopes and intercepts of all analytes include 1 and 0, respectively (see the Supplementary Material for more details). Notably in the electrolyte panel (Na, Cl, and K), there were several data points deviated from ±2*SD of the mean difference in the Bland–Altman plots (Figure 2E–H). Such deviations were generally restricted to the electrolyte panel results from one of the two chemistry analyzers. Note that results for Na, Cl, and K should be interpreted with caution. Table 2 shows a summary of mean % bias for each analyte at each site and for each chemistry analyzer that was used (see Supplementary Tables S3–S16 for more details). The mean % biases across all sites and analyzers were well below clinically significant levels (i.e., desirable biological variation specification) taken from the Westgard QC [29], which was first published in 1999 by Ricos et al. [30] and updated in 2014: −1.02% for K, 0.36% for Na, 0.34% for Cl, and 0.26% for Ca in the electrolyte panel; −1.71% for AST, 2.27% for ALT, 1.20% for ALP, 0.45% for total bilirubin, 1.71% for albumin, and 0.58% for total protein in the liver panel; −0.02% for BUN and 1.65% for creatinine in the kidney panel; and 0.34% for Chol and 0.58% for TRIG in the lipid panel.

### 3.2. Precision

Table 3 shows the %CV, and components of variance for the lot-to-lot precision and the inter-operator precision across the sites. The total variances for both lot-to-lot precision and inter-operator precision were within the acceptable limits for all analytes: K (2.89%), Na (0.72%), Cl (0.81%), Ca (0.93%), BUN (2.47%), Cre (2.75%), AST (6.41%), ALT (6.93%), ALP (1.81%), T.Bili (4.19%), Alb (2.25%), TP (1.63%), Chol (1.88%), and TRIG (2.18%) across different production lots, and K (2.87%), Na (0.67%), Cl (0.88%), Ca (1.69%), BUN (2.23%), Cre (2.82%), AST (6.50%), ALT (6.19%), ALP (1.66%), T. Bili (4.21%), Alb (2.02%), TP (1.68%), Chol (1.81%), and TRIG (1.99%) among different operators within a single production lot. The precision results for each analyte were comparable for each of the three clinical sites as shown in the Supplementary Material (Supplementary Tables S17–S20 for the lot-to-lot precision results and Supplementary Tables S21–S24 for the inter-operator precision results).

### 3.3. Plasma Quality

Blood cell counts and hemoglobin levels in plasma processed in ZDiscs are shown in Table 4. The average hemoglobin concentration was 8.03 mg/dL, which is ~10-fold below the acceptable threshold of 100 mg/dL. The average cell counts were 1.3 × 10^6^ RBC/mL, 0.045 × 10^6^ WBC/mL, and 2.8 × 10^6^ PLT/mL, again falling well below the acceptance thresholds.

## 4. Discussion

The study findings demonstrate the equivalence of the ZDisc to the PST when used by healthcare professionals to collect and process venous blood. The 134-patient performance comparison study showed that the ZDisc and PST yield equivalent measurements for all 14 analytes. Mean percent biases between the ZDisc and PST were not significant based on each analyte’s clinically significant levels for any analyte. Furthermore, the 31-patient precision study demonstrated that the ZDisc meets the reproducibility acceptance criteria for clinical laboratory measurement of target analytes in plasma, and assessments of plasma quality support the suitability of the ZDisc to produce high quality plasma for clinical laboratory analysis.

One limitation of this study is that diluted samples were used to augment the range of analytes found in subjects. Although dilution was limited (i.e., cases less than 5% were diluted in the performance comparison study and less than 40% for the precision study), it is possible that the dilution process could cause distortions in assay methods, such as changing the background signal in spectrophotometric methods used to measure AST, ALT, and ALP. It is expected that such distortion would also be found in the control tubes. Furthermore, it is of note that native samples are preferable for the performance comparison study, rather than modified samples. Unfortunately, recruiting subjects with severe but transient health conditions associated with extreme high or low electrolyte concentrations proved impractical. Therefore, the results for Na, Cl, and K from the modified samples should be interpreted with caution.

Another limitation is that frozen samples were used for logistical purposes, so that two different chemistry laboratories could be used, for which we had time-limited access. It is possible that the freeze–thaw process and prolonged storage of frozen samples may induce artifacts into the analytes’ concentrations; however, we believe that the results of our studies with thawed plasma samples will translate to samples maintained as unfrozen liquid [16,31].

The Torq system (ZDisc and ZDrive) is intended to expand decentralized blood sampling access by bringing specimen centrifugation to the point of collection. Prompt centrifugation is especially important for certain analytes such as potassium, bilirubin, creatinine, and particular liver enzymes. For example, potassium is abundant within blood cells, where deterioration of the cells in whole blood prior to plasma separation can falsely elevate analyte concentration in plasma, and subsequently negate the reliability of the results [32,33]. Bilirubin, creatinine, ALT, and AST have also been shown to be unstable in whole blood when plasma separation is delayed [16,34,35].

In addition to the improvements in sample quality and analytical accuracy, Torq technology provides operational workflow advantages by enabling a compact, pocket-size, and battery-powered centrifuge system easily transported to any remote sampling settings such as a pop-up clinic, workplace, or home. ZDiscs are loaded using conventional phlebotomy supplies, and can be loaded sequentially with conventional blood tubes without a separate venipuncture. Lastly, centrifuging blood samples at the point of collection immensely improves laboratory efficiencies and turnaround times by negating the need for upfront centrifugation upon sample arrival; instead, samples can be directly routed for testing upon arrival at the laboratory.

## Figures and Tables

**Table 1:** Primary upper and lower medical decision limits as applicable for all analytes tested.

Analyte	Lower MDL	Upper MDL
K	3.5 mmol/L	5.5 mmol/L
Na	130 mmol/L	150 mmol/L
Cl	95 mmol/L	109 mmol/L
Ca	8.6 mg/dL	10.3 mg/dL
BUN	6 mg/dL	25 mg/dL
Cre	1.5 mg/dL	0.4 mg/dL
AST	N/A	35 U/L
ALT	N/A	35 U/L
ALP	N/A	120 U/L
T.Bili	0.3 mg/dL	1.5 mg/dL
Alb	3.5 g/dL	5.5 g/dL
TP	5.5 g/dL	9.0 g/dL
Chol	N/A	200 mg/dL
TRIG	N/A	150 mg/dL

**Table 2:** Mean percent bias for all analytes at each site and for each chemistry analyzer.

Site/Analyzer		K	Na	Cl	Ca	AST	ALT	ALP
Site 1	Analyzer 1	−0.648	1.264	0.991	1.445	−0.386	2.783	2.783
	Analyzer 2	−1.866	−0.082	−0.379	−0.445	−0.387	0.517	0.517
	Analyzer Pooled	−1.257	0.591	0.306	0.500	−0.387	1.650	1.650
Site 2	Analyzer 1	−0.339	0.449	0.477	0.957	−1.286	2.195	2.195
	Analyzer 2	−0.870	0.206	0.320	0.042	−1.582	0.752	0.752
	Analyzer Pooled	−0.604	0.328	0.399	0.499	−1.434	1.474	1.474
Site 3	Analyzer 1	−1.748	0.002	0.175	−0.406	−4.234	0.147	0.147
	Analyzer 2	−0.728	0.256	0.451	−0.241	−3.182	0.501	0.501
	Analyzer Pooled	−1.238	0.129	0.313	−0.323	−3.708	0.324	0.324
3 sites pooled/Analyzer 1		−0.857	0.605	0.570	0.722	−1.810	4.328	1.805
3 sites pooled/Analyzer 2		−1.178	0.120	0.113	−0.208	−1.617	0.212	0.597
Total		−1.018	0.362	0.341	0.257	−1.714	2.270	1.201
**Site/Analyzer**		**Total Bilirubin**	**Albumin**	**Total Protein**	**BUN**	**Creatinine**	**Cholesterol**	**Triglycerides**
Analyzer 1		2.206	2.878	1.979	0.665	1.875	N/A	N/A
Analyzer 2		0.593	1.290	0.541	0.525	1.808	0.331	1.038
Analyzer Pooled		1.400	2.084	1.260	0.595	1.841	0.331	1.038
Analyzer 1		3.971	2.166	1.187	−0.730	1.929	N/A	N/A
Analyzer 2		−0.299	1.296	−0.326	0.093	1.821	0.416	0.038
Analyzer Pooled		1.836	1.731	0.431	−0.319	1.875	0.416	0.038
Analyzer 1		−2.452	1.141	0.137	−0.999	0.787	N/A	N/A
Analyzer 2		−2.488	1.297	−0.249	0.245	1.504	0.259	0.680
Analyzer Pooled		−2.470	1.219	−0.056	−0.377	1.146	0.259	0.680
3 sites pooled/Analyzer 1		1.513	2.119	1.153	−0.319	1.575	N/A	N/A
3 sites pooled/Analyzer 2		−0.616	1.294	−0.003	0.289	1.723	0.340	0.575
Total		0.449	1.706	0.575	−0.015	1.649	0.340	0.575

**Table 3:** Total percent (%) CV and components of variance between lot/operator and within lot/operator across all the sites. K: Potassium; Na: Sodium; Cl: Chloride; Ca: Calcium; BUN: Blood Urea Nitrogen; Cre: Creatinine; AST: Aspartate Aminotransferase; ALT: Alanine Aminotransferase; ALP: Alkaline Phosphatase; T.Bili: Total Bilirubin; Alb: Albumin; TP: Total Protein; Chol: Cholesterol; TRIG: Triglycerides. Note that 0.00 * is negative ANOVA-type estimated CVs which were set to 0.00, and which did not contribute to the total variance.

Analyte (Sample Size, *n*)	Lot-to-Lot Precision Mean % CV (95% Confidence Interval)			Inter-operator Precision Mean % CV (95% Confidence Interval)		
	Between Lot	Within Lot	Total	Between Operator	Within Operator	Total
K (mmol/L) (*n* = 171)	0.63 (0.00–1.21)	2.82 (2.52–3.19)	2.89 (2.56–3.31)	0.83 (0.00–1.38)	2.75 (2.46–3.10)	2.87 (2.55–3.27)
Na (mmol/L) (*n* = 171)	0.09 (0.00–0.21)	0.72 (0.64–0.71)	0.72 (0.65–0.82)	0.06 (0.00–0.18)	0.67 (0.60–0.76)	0.67 (0.61–0.76)
Cl (mmol/L) (*n* = 171)	0.15 (0.00–0.30)	0.80 (0.71–0.91)	0.81 (0.72–0.93)	0.05 (0.00–0.22)	0.88 (0.79–0.99)	0.88 (0.79–0.99)
Ca (mg/dL) (*n* = 166)	0.22 (0.00–0.42)	0.91 (0.81–1.03)	0.93 (0.83–1.08)	0.18 (0.00–0.49)	1.68 (1.50–1.91)	1.69 (1.51–1.91)
BUN (mg/dL) (*n* = 168)	0.34 (0.00–0.75)	2.45 (2.19–2.77)	2.47 (2.20–2.81)	0.27 (0.00–0.66)	2.21 (1.98–2.50)	2.23 (2.00–2.52)
Cre (mg/dL) (*n* = 165)	0.14 (0.00–0.58)	2.75 (2.45–3.12)	2.75 (2.46–3.12)	0.00 *	2.82 (2.52–3.19)	2.82 (2.53–3.18)
AST (U/L) (*n* = 173)	2.11 (0.00–3.80)	6.06 (5.42–6.86)	6.41 (5.54–7.62)	1.89 (0.00–3.12)	6.22 (5.59–7.02)	6.50 (5.79–7.41)
ALT (U/L) (*n* = 172)	1.04 (0.00–2.21)	6.85 (6.13–7.76)	6.93 (6.19–7.87)	0.96 (0.00–2.01)	6.12 (5.49–6.91)	6.19 (5.56–6.98)
ALP (U/L) (*n* = 174)	0.41 (0.00–0.78)	1.76 (1.58–1.99)	1.81 (1.60–2.07)	0.74 (0.00–1.13)	1.48 (1.33–1.68)	1.66 (1.43–1.97)
T.Bili (mg/dL) (*n* = 163)	0.62 (0.00–1.33)	4.15 (3.70–4.72)	4.19 (3.73–4.78)	0.61 (0.00–1.36)	4.16 (3.72–4.73)	4.21 (3.76–4.77)
Alb (mg/dL) (*n* = 170)	0.85 (0.00–1.52)	2.08 (1.86–2.36)	2.25 (1.90–2.76)	0.74 (0.00–1.17)	1.88 (1.68–2.12)	2.02 (1.77–2.34)
TP (mg/dL) (*n* = 169)	0.61 (0.00–1.08)	1.51 (1.35–1.71)	1.63 (1.38–1.99)	0.80 (0.00–1.23)	1.48 (1.32–1.67)	1.68 (1.43–2.03)
Chol (mg/dL) (*n* = 170)	0.36 (0.00 *–0.72)	1.85 (1.65–2.09)	1.88 (1.68–2.15)	0.82 (0.00 *–1.26)	1.61 (1.45–1.82)	1.81 (1.55–2.16)
TRIG (mg/dL) (*n* = 170)	0.00 *	2.18 (1.95–2.47)	2.18 (1.95–2.47)	0.13 (0.00 *–0.51)	1.99 (1.78–2.25)	1.99 (1.79–2.25)

**Table 4:** Average blood cell count and hemoglobin concentration in plasma from the ZDiscs. RBC: Red Blood Cell; WBC: White Blood Cell; PLT: Platelet; CI: Confidence Interval.

Parameters	RBC (M/mL) Threshold: 6 M/mL	WBC (M/mL) Threshold: 0.1 M/mL	PLT (M/mL) Threshold: 50 M/mL	Mean Hb (mg/dL) Threshold: 100 mg/mL
Average	1.30	0.045	2.79	8.03
95% CI	1.08–1.52	0.035–0.056	1.91–3.67	6.44–9.62

## Supplementary Materials

The following are available online at https://www.mdpi.com/article/10.3390/diagnostics11061019/s1, Figure S1. Modified blood collection set including 21G butterfly 12-inch tubing to transfer the modified samples in a 50 mL conical tube into a ZDisc, Table S1: A summary of subject demographic statistics, Table S2: Extreme MDLs used for mean percent bias analysis tested with common pathophysiology associated with extreme MDL values, Table S3: A summary of performance comparison results for potassium (K), Table S4: A summary of performance comparison results for sodium (Na), Table S5: A summary of performance comparison results for chloride (Cl), Table S6: A summary of performance comparison results for calcium (Ca), Table S7: A summary of performance comparison results for Aspartate Aminotransferase (AST), Table S8: A summary of performance comparison results for Alanine Aminotransferase (ALT), Table S9: A summary of performance comparison results for Alkaline Phosphatase (ALP), Table S10: A summary of performance comparison results for total bilirubin (T.Bili), Table S11: A summary of performance comparison results for albumin (Alb), Table S12: A summary of performance comparison results for total protein (TP), Table S13: A summary of performance comparison results for blood urea nitrogen (BUN), Table S14: A summary of performance comparison results for creatinine (Cre), Table S15: A summary of performance comparison results for cholesterol (Chol), Table S16: A summary of performance comparison results for triglycerides (TRIG), Table S17: Total percent (%) CV and components of variance between lots and within lots based on site and analyte for electrolytes, Table S18: Total percent (%) CV and components of variance between lots and within lots based on site and analyte for liver function tests, Table S19: Total percent (%) CV and components of variance between lots and within lots based on site and analyte for kidney function tests, Table S20: Total percent (%) CV and components of variance between lots and within lots based on site and analyte for lipid tests, Table S21: Total percent (%) CV and components of variance between operators and within operators based on site and analyte for electrolytes, Table S22: Total percent (%) CV and components of variance between operators and within operators based on site and analyte for liver function tests, Table S23: Total percent (%) CV and components of variance between operators and within operators based on site and analyte for kidney function tests, Table S24: Total percent (%) CV and components of variance between operators and within operators based on site and analyte for lipid tests, Table S25: Grand mean with SD and mean % difference between the direct draw group vs. the contrived group for each analyte.
