# Supplementary material for: Clinical Evaluation of the Torq Zero Delay Centrifuge System for Decentralized Blood Collection and Stabilization
Source: Diagnostics (Basel). 2021 Jun 2;11(6):1019. doi: 10.3390/diagnostics11061019 (PMC8226604; doi:10.3390/diagnostics11061019)
Supplement: Supplementary file 1 [file diagnostics-11-01019-s001.zip › diagnostics-1228046-supplementary.pdf]

Table S1. A summary of subject demographic statistics

Demographic Parameters		Sample size (n)	Percent (%)
**Age**	Mean +/- SD	50.76 +/- 16.25	N/A
	Median	54.50	N/A
	Min	18	N/A
	Max	79	N/A
**Gender**	Male	27	20.15
	Female	105	78.36
	Declined to answer	2	1.49
**Race**	White	93	69.40
	Hispanic or Latino	20	14.93
	Black or African American	15	11.19
	Native American or American Indian	0	0.00
	Asian or Pacific Islander	1	0.75
	Others	5	3.73
**Total**		**134**	N/A

Table S2. Extreme MDLs used for mean percent bias analysis tested with common pathophysiology associated with extreme MDL values.

Analyte	Extreme MDL	Common Pathophysiology of Extreme MDL
K	≥ 7 mmol/L	Severe hyperkalemia in the case of renal failure or blood transfusion
Na	≤ 115 mmol/L	Severe hyponatremia in the case of burns or hypotonic IV use
AST	≥ 300 U/L	Infectious hepatitis, primary or metastatic carcinoma of the liver and other inflammatory conditions affecting the liver
ALT	≥ 250 U/L	Infectious hepatitis, primary or metastatic carcinoma of the liver and other inflammatory conditions affecting the liver
ALP	≥ 300 U/L	Extra/intrahepatic obstruction (e.g. by stone or cancer)

Table S3. A summary of performance comparison results for potassium (K)

Site	Sample Size (n)	Analyzer	Slope (95% CI)	Intercept (95% CI)	Pearson's coefficient	Mean % Bias

**Site 1**	**47**	**Analyzer 1**	1.025<br>(0.960 – 1.163)	-0.075<br>(-0.614 - 0.195)	0.954	-0.648
	**47**	**Analyzer 2**	1.062<br>(0.981 - 1.273)	-0.181<br>(-1.061 - 0.147)	0.940	-1.866
	**94**	**Analyzer Pooled**	1.047<br>(0.992 – 1.162)	-0.145<br>(-0.618 – 0.086)	0.946	-1.257
**Site 2**	**48**	**Analyzer 1**	0.960<br>(0.846 - 1.053)	0.170<br>(-0.199 - 0.612)	0.929	-0.339
	**48**	**Analyzer 2**	0.956<br>(0.870 - 1.112)	0.211<br>(-0.407 - 0.547)	0.924	-0.870
	**96**	**Analyzer Pooled**	0.963<br>(0.894 – 1.046)	0.170<br>(-0.169 – 0.435)	0.927	-0.604
**Site 3**	**39**	**Analyzer 1**	0.993<br>(0.870 - 1.142)	0.096<br>(-0.509 - 0.587)	0.910	-1.748
	**39**	**Analyzer 2**	0.929<br>(0.811 - 1.071)	0.316<br>(-0.241 - 0.798)	0.909	-0.728
	**78**	**Analyzer Pooled**	0.949<br>(0.864 – 1.050)	0.254<br>(-0.150 – 0.598)	0.908	-1.238
	**Pooled (n=134)**		1.027<br>(0.982 - 1.102)	-0.069<br>(-0.355 - 0.111)	0.944	-1.018

Table S4. A summary of performance comparison results for sodium (Na)

Site	Sample Size (n)	Analyzer	Slope (95% CI)	Intercept (95% CI)	Pearson’s coefficient	Mean % Bias
**Site 1**	**47**	**Analyzer 1**	0.881<br>(0.739 – 0.949)	14.971<br>(5.892 – 35.446)	0.946	1.264
	**47**	**Analyzer 2**	0.984<br>(0.923 – 1.046)	2.290<br>(-6.298 – 10.864)	0.981	-0.082
	**94**	**Analyzer Pooled**	0.916<br>(0.842 – 0.963)	10.975<br>(4.384 – 21.169)	0.956	0.591
**Site 2**	**48**	**Analyzer 1**	0.606<br>(0.441 – 0.854)	54.527<br>(19.674 – 77.674)	0.835	0.449
	**48**	**Analyzer 2**	0.984	1.919	0.859	0.206

			(0.837 - 1.147)	(-21.100 – 22.980)		
	**96**	**Analyzer Pooled**	0.799 (0.651 – 0.976)	27.824 (2.932 – 48.503)	0.837	0.328
**Site 3**	**39**	**Analyzer 1**	1.083 (0.902 - 1.286)	-11.599 (-39.832 – 13.630)	0.898	0.002
	**39**	**Analyzer 2**	0.925 (0.738 – 1.112)	10.169 (-15.819 – 36.126)	0.844	0.256
	**78**	**Analyzer Pooled**	1.015 (0.891 – 1.149)	-2.324 (-20.934 – 15.017)	0.869	0.129
	**Pooled (n=134)**		0.911 (0.840 – 0.960)	11.934 (5.226 – 21.825)	0.937	0.362

Table S5. A summary of performance comparison results for chloride (Cl)

**Site**	**Sample Size (n)**	**Analyzer**	**Slope (95% CI)**	**Intercept (95% CI)**	**Pearson's coefficient**	**Mean % Bias**
**Site 1**	**47**	**Analyzer 1**	0.944 (0.838 – 1.006)	4.848 (-1.512 – 15.437)	0.970	0.991
	**47**	**Analyzer 2**	0.993 (0.947 – 1.045)	1.081 (-4.159 – 5.873)	0.986	-0.379
	**94**	**Analyzer Pooled**	0.957 (0.902 – 0.996)	4.117 (0.348 – 9.696)	0.975	0.306
**Site 2**	**48**	**Analyzer 1**	0.896 (0.783 – 1.019)	10.322 (-2.514 – 22.123)	0.881	0.477
	**48**	**Analyzer 2**	0.974 (0.856 – 1.124)	2.408 (-13.141 – 14.750)	0.906	0.320
	**96**	**Analyzer Pooled**	0.937 (0.854 – 1.029)	6.110 (-3.310 – 14.654)	0.893	0.399
**Site 3**	**39**	**Analyzer 1**	1.027 (0.946 – 1.125)	-2.982 (-13.217 – 5.495)	0.948	0.175
	**39**	**Analyzer 2**	0.977 (0.786 – 1.123)	1.869 (-12.927 – 21.573)	0.889	0.451
	**78**	**Analyzer Pooled**	1.017 (0.930 – 1.096)	-2.020 (-10.167 – 6.832)	0.920	0.313

**Pooled (n=134)**			0.959 (0.913 – 0.992)	3.869 (0.565 – 8.567)	0.964	0.341

Table S6. A summary of performance comparison results for calcium (Ca)

Site	Sample Size (n)	Analyzer	Slope (95% CI)	Intercept (95% CI)	Pearson's coefficient	Mean % Bias
**Site 1**	**44**	**Analyzer 1**	0.931 (0.866 - 1.005)	0.508 (-0.165 – 1.094)	0.962	1.445
	**44**	**Analyzer 2**	1.007 (0.960 - 1.082)	-0.024 (-0.731 – 0.404)	0.986	-0.445
	**88**	**Analyzer Pooled**	0.958 (0.914 – 1.001)	0.338 (-0.026 – 0.721)	0.966	0.500
**Site 2**	**45**	**Analyzer 1**	0.963 (0.823 – 1.147)	0.248 (-1.455 – 1.562)	0.871	0.957
	**45**	**Analyzer 2**	0.955 (0.869 - 1.031)	0.415 (-0.296 – 1.243)	0.945	0.042
	**90**	**Analyzer Pooled**	0.995 (0.896 – 1.096)	0.001 (-0.951– 0.926)	0.903	0.499
**Site 3**	**38**	**Analyzer 1**	0.880 (0.759 – 0.980)	1.174 (0.249 – 2.296)	0.922	-0.406
	**38**	**Analyzer 2**	1.116 (0.966 – 1.243)	-1.070 (-2.263 – 0.342)	0.917	-0.241
	**76**	**Analyzer Pooled**	0.978 (0.880 – 1.072)	0.234 (-0.647 – 1.151)	0.914	-0.323
	**Pooled (n=127)**		0.974 (0.935 – 1.010)	0.218 (-0.118 – 0.573)	0.951	0.257

Table S7. A summary of performance comparison results for Aspartate Aminotransferase (AST)

Site	Sample Size (n)	Analyzer	Slope (95% CI)	Intercept (95% CI)	Pearson's coefficient	Mean % Bias
**Site 1**	**47**	**Analyzer 1**	1.009	-0.179	0.999	-0.386

			(0.868 - 1.030)	(-0.745 – 2.356)		
	47	Analyzer 2	1.002<br>(0.972 - 1.044)	0.027<br>(-0.844 – 0.680)	1.000	-0.387
	94	Analyzer Pooled	1.005<br>(0.977 – 1.023)	-0.066<br>(-0.462 – 0.462)	1.000	-0.387
Site 2	48	Analyzer 1	0.942<br>(0.869 – 0.974)	1.354<br>(0.463 – 2.730)	0.965	-1.286
	48	Analyzer 2	0.979<br>(0.887 - 1.016)	0.800<br>(-0.358 – 2.806)	0.962	-1.582
	96	Analyzer Pooled	0.965<br>(0.909 – 0.993)	0.998<br>(0.212 – 2.179)	0.965	-1.434
Site 3	38	Analyzer 1	0.992<br>(0.980 - 1.121)	0.964<br>(-1.114 – 1.511)	0.996	-4.234
	38	Analyzer 2	0.994<br>(0.982 - 1.065)	0.849<br>(-0.746 – 1.374)	0.998	-3.182
	76	Analyzer Pooled	0.993<br>(0.984 – 1.052)	0.908<br>(-0.250 – 1.254)	0.997	-3.708
	Pooled (n=133)		1.003<br>(0.982 – 1.018)	0.236<br>(-0.140 – 0.726)	0.999	-1.714

Table S8. A summary of performance comparison results for Alanine Aminotransferase (ALT)

Site	Sample Size (n)	Analyzer	Slope (95% CI)	Intercept (95% CI)	Pearson's coefficient	Mean % Bias
Site 1	46	Analyzer 1	0.997<br>(0.968 – 0.998)	-0.437<br>(-0.629 – -0.034)	1.000	3.870
	46	Analyzer 2	0.987<br>(0.977 - 1.063)	0.424<br>(-0.742 – 1.032)	0.999	-1.811
	92	Analyzer Pooled	0.992<br>(0.982 – 1.001)	0.002<br>(-0.423 – 0.365)	0.999	1.029
Site 2	48	Analyzer 1	0.975<br>(0.930 – 1.016)	-0.241<br>(-0.821 – 0.347)	0.991	4.356
	48	Analyzer 2	1.002<br>(0.972 - 1.037)	-0.294<br>(-0.777 – 0.097)	0.995	2.444

	**96**	**Analyzer Pooled**	0.985 (0.957 – 1.013)	-0.229 (-0.599– 0.136)	0.992	3.400
**Site 3**	**36**	**Analyzer 1**	0.876 (0.823 – 0.967)	0.887 (-0.215 – 1.500)	0.994	4.875
	**36**	**Analyzer 2**	0.918 (0.771 – 0.971)	0.769 (0.097 – 2.313)	0.982	-0.180
	**72**	**Analyzer Pooled**	0.896 (0.834 – 0.959)	0.839 (0.125 – 1.559)	0.987	2.348
	**Pooled (n=130)**		0.987 (0.967 – 0.996)	-0.197 (-0.400 – 0.107)	0.998	2.270

Table S9. A summary of performance comparison results for Alkaline Phosphatase (ALP)

Site	Sample Size (n)	Analyzer	Slope (95% CI)	Intercept (95% CI)	Pearson's coefficient	Mean % Bias
**Site 1**	**47**	**Analyzer 1**	0.983 (0.948 - 1.008)	-0.850 (-3.020 – 1.701)	0.999	2.783
	**47**	**Analyzer 2**	0.993 (0.970 - 1.004)	0.074 (-0.865 – 1.848)	1.000	0.517
	**94**	**Analyzer Pooled**	0.988 (0.975 – 1.003)	-0.320 (-1.722 – 0.804)	0.999	1.650
**Site 2**	**48**	**Analyzer 1**	0.963 (0.935 – 1.015)	1.096 (-2.387 – 3.193)	0.995	2.195
	**48**	**Analyzer 2**	0.994 (0.980 - 1.020)	-0.059 (-1.741 – 0.899)	0.999	0.752
	**96**	**Analyzer Pooled**	0.982 (0.957 – 1.010)	0.331 (-1.667– 2.031)	0.996	1.474
**Site 3**	**39**	**Analyzer 1**	0.994 (0.961 - 1.021)	0.332 (-1.442 – 2.491)	0.997	0.147
	**39**	**Analyzer 2**	0.966 (0.928 – 0.998)	1.954 (-0.190 – 4.505)	0.996	0.501
	**78**	**Analyzer Pooled**	0.978 (0.951 – 1.000)	1.259 (-0.248 – 3.039)	0.996	0.324
	**Pooled (N=134)**		0.984	0.299	0.998	1.201

	(0.974 – 0.995)	(-0.529 – 1.018)		

Table S10. A summary of performance comparison results for total bilirubin (T.Bili)

Site	Sample Size (n)	Analyzer	Slope (95% CI)	Intercept (95% CI)	Pearson's coefficient	Mean % Bias
Site 1	45	Analyzer 1	1.045 (0.940 – 1.089)	-0.015 (-0.053 - 0.014)	0.946	2.206
	45	Analyzer 2	1.027 (0.981 - 1.073)	-0.010 (-0.024 - 0.007)	0.988	0.593
	90	Analyzer Pooled	1.021 (0.971 – 1.051)	-0.010 (-0.026 – 0.006)	0.969	1.400
Site 2	47	Analyzer 1	0.907 (0.823 – 0.991)	0.022 (-0.012 - 0.053)	0.971	3.971
	47	Analyzer 2	0.993 (0.966 - 1.015)	0.003 (-0.008 - 0.015)	0.996	-0.299
	94	Analyzer Pooled	0.946 (0.892 – 0.992)	0.013 (-0.005 – 0.032)	0.983	1.836
Site 3	37	Analyzer 1	1.030 (0.964 - 1.136)	-0.002 (-0.039 - 0.027)	0.963	-2.452
	37	Analyzer 2	1.055 (1.005 - 1.094)	-0.010 (-0.027 - 0.008)	0.989	-2.488
	74	Analyzer Pooled	1.004 (1.000 – 1.093)	-0.006 (-0.024 – 0.011)	0.977	-2.470
	Pooled (n=129)		0.978 (0.944 - 1.010)	0.007 (-0.005 - 0.018)	0.976	0.449

Table S11. A summary of performance comparison results for albumin (Alb)

Site	Sample Size (n)	Analyzer	Slope (95% CI)	Intercept (95% CI)	Pearson's coefficient	Mean % Bias
Site 1	**46**	**Analyzer 1**	0.917 (0.820 - 1.040)	0.232 (-0.309 – 0.639)	0.910	2.878
	**46**	**Analyzer 2**	0.952 (0.883 - 1.038)	0.152 (-0.221 – 0.452)	0.961	1.290
	**92**	**Analyzer Pooled**	0.941 (0.882 – 1.010)	0.162 (-0.136 – 0.422)	0.930	2.084
Site 2	**48**	**Analyzer 1**	1.021 (0.827 – 1.278)	-0.177 (-1.230 – 0.634)	0.821	2.166
	**48**	**Analyzer 2**	1.025 (0.934 - 1.177)	-0.167 (-0.823 – 0.225)	0.948	1.296
	**96**	**Analyzer Pooled**	1.047 (0.951 – 1.174)	-0.276 (-0.819 – 0.145)	0.903	1.731
Site 3	**38**	**Analyzer 1**	0.890 (0.776 - 1.006)	0.420 (-0.067 – 0.894)	0.899	1.141
	**38**	**Analyzer 2**	1.000 (0.870 - 1.208)	-0.057 (-0.988 – 0.528)	0.905	1.297
	**76**	**Analyzer Pooled**	0.949 (0.880 – 1.041)	0.171 (-0.242 – 0.466)	0.916	1.219
	**Pooled (n=132)**		0.978 (0.934 – 1.023)	0.021 (-0.179 – 0.214)	0.919	1.706

Table S12. A summary of performance comparison results for total protein (TP)

Site	Sample Size (n)	Analyzer	Slope (95% CI)	Intercept (95% CI)	Pearson's coefficient	Mean % Bias
Site 1	**46**	**Analyzer 1**	0.874 (0.774 – 0.951)	0.760 (0.224 – 1.467)	0.927	1.979
	**46**	**Analyzer 2**	1.031 (0.966 – 1.101)	-0.264 (-0.744 – 0.193)	0.973	0.541
	**92**	**Analyzer Pooled**	0.962 (0.911 – 1.014)	0.179 (-0.179 – 0.526)	0.943	1.260
Site 2	**48**	**Analyzer 1**	0.962	0.180	0.892	1.187

			(0.792 – 1.104)	(-0.789 – 1.363)		
	**48**	**Analyzer 2**	0.996 (0.920 - 1.075)	0.050 (-0.520 – 0.585)	0.962	-0.326
	**96**	**Analyzer Pooled**	0.999 (0.907 – 1.085)	-0.024 (-0.634 – 0.613)	0.923	0.431
**Site 3**	**39**	**Analyzer 1**	0.981 (0.840 - 1.089)	0.130 (-0.629 – 1.160)	0.907	0.137
	**39**	**Analyzer 2**	1.123 (0.997 – 1.290)	-0.896 (-2.162 – 0.057)	0.913	-0.249
	**78**	**Analyzer Pooled**	1.056 (0.964 – 1.141)	-0.407 (-1.038 – 0.266)	0.909	-0.056
	**Pooled (n=133)**		1,003 (0.964 – 1.045)	-0.063 (-0.368 – 0.225)	0.933	0.575

Table S13. A summary of performance comparison results for blood urea nitrogen (BUN)

Site	Sample Size (n)	Analyzer	Slope (95% CI)	Intercept (95% CI)	Pearson's coefficient	Mean % Bias
**Site 1**	**47**	**Analyzer 1**	1.011 (0.966 - 1.058)	-0.233 (-0.838 – 0.353)	0.992	0.665
	**47**	**Analyzer 2**	0.999 (0.973 - 1.021)	-0.044 (-0.379 – 0.301)	0.995	0.525
	**94**	**Analyzer Pooled**	1.005 (0.979 – 1.028)	-0.140 (-0.460 – 0.189)	0.994	0.595
**Site 2**	**48**	**Analyzer 1**	0.984 (0.959 – 1.016)	0.317 (-0.134 – 0.689)	0.995	-0.730
	**48**	**Analyzer 2**	0.998 (0.985 - 1.012)	0.008 (-0.208 – 0.217)	0.997	0.093
	**96**	**Analyzer Pooled**	0.991 (0.977 – 1.007)	0.156 (-0.080 – 0.389)	0.996	-0.319
**Site 3**	**39**	**Analyzer 1**	1.032 (0.975 - 1.084)	-0.277 (-0.998 – 0.501)	0.991	-0.999
	**39**	**Analyzer 2**	0.996 (0.964 - 1.032)	0.033 (-0.412 – 0.423)	0.996	0.245

Site	Sample Size (n)	Analyzer	Slope (95% CI)	Intercept (95% CI)	Pearson's coefficient	Mean % Bias
	**78**	**Analyzer Pooled**	1.015 (0.979 – 1.051)	-0.132 (-0.577 – 0.315)	0.993	-0.377
	**Pooled (n=134)**		1.001 (0.990 – 1.016)	-0.014 (-0.209 – 0.152)	0.995	-0.015

Table S14. A summary of performance comparison results for creatinine (Cre)

Site	Sample Size (n)	Analyzer	Slope (95% CI)	Intercept (95% CI)	Pearson's coefficient	Mean % Bias
**Site 1**	**46**	**Analyzer 1**	0.939 (0.905 – 0.977)	0.030 (0.001 – 0.055)	0.989	1.875
	**46**	**Analyzer 2**	0.980 (0.919 - 1.047)	0.001 (-0.046 – 0.043)	0.988	1.808
	**92**	**Analyzer Pooled**	0.960 (0.926 – 1.001)	0.014 (-0.012 – 0.039)	0.988	1.841
**Site 2**	**48**	**Analyzer 1**	0.973 (0.953 – 1.008)	0.007 (-0.026 – 0.027)	0.994	1.929
	**48**	**Analyzer 2**	0.997 (0.956 - 1.026)	-0.012 (-0.035 – 0.019)	0.997	1.821
	**96**	**Analyzer Pooled**	0.985 (0.961 – 1.011)	-0.003 (-0.024– 0.017)	0.995	1.875
**Site 3**	**39**	**Analyzer 1**	0.993 (0.923 – 1.051)	-0.001 (-0.046 – 0.053)	0.984	0.787
	**39**	**Analyzer 2**	1.011 (0.941 – 1.080)	-0.019 (-0.073 – 0.033)	0.981	1.504
	**78**	**Analyzer Pooled**	0.969 (0.949 – 0.991)	0.012 (-0.005 – 0.027)	0.982	1.146
	**Pooled (n=133)**		0.982 (0.964 – 1.001)	0.001 (-0.012 – 0.015)	0.992	1.649

Table S15. A summary of performance comparison results for cholesterol (Chol)

Site	Sample Size (n)	Analyzer	Slope (95% CI)	Intercept (95% CI)	Pearson's coefficient	Mean % Bias
**Site 1**	**47**	**Analyzer 2**	0.963 (0.934 - 1.005)	5.876 (-1.651 – 11.038)	0.996	-0.719
**Site 2**	**48**	**Analyzer 2**	0.998 (0.983 - 1.010)	-0.365 (-2.943 – 2.395)	0.999	-0.401
**Site 3**	**39**	**Analyzer 2**	0.999 (0.960 – 1.037)	-0.399 (-7.485 – 6.518)	0.994	-0.260
	**Pooled (n=133)**		0.988 (0.972 – 1.004)	1.426 (-1.336 – 4.287)	0.997	-0.438

Table S16. A summary of performance comparison results for triglycerides (TRIG)

Site	Sample Size (n)	Analyzer	Slope (95% CI)	Intercept (95% CI)	Pearson's coefficient	Mean % Bias
**Site 1**	**47**	**Analyzer 2**	0.995 (0.972 – 1.015)	-0.857 (-2.751 – 1.154)	0.997	-1.057
**Site 2**	**48**	**Analyzer 2**	0.997 (0.978 – 1.020)	0.200 (-2.349 – 2.289)	0.998	-0.121
**Site 3**	**39**	**Analyzer 2**	1.064 (0.970 – 1.105)	-7.902 (-12.417 – 1.805)	0.997	1.160
	**Pooled (n=133)**		1.017 (0.986 – 1.050)	-2.856 (-6.932 – 0.676)	0.997	-0.251

Table S17. Total percent (%) CV and components of variance between lots and within lots based on site and analyte for electrolytes.

Analyte (Unit)	Variance Component	Mean % CV		
		**Site 1**	**Site 2**	**Site 3**
K (mmol/L)	Between Lot	0.00 *	0.64	0.25
	Within Lot	4.23	1.93	1.83

	Total	4.23	2.03	1.84
	Mean (sample size)	4.47 (n = 56)	4.71 (n = 55)	4.41 (n = 60)
Na (mmol/L)	Between Lot	0.42	0.00 *	0.00 *
	Within Lot	0.65	0.66	0.76
	Total	0.77	0.66	0.76
	Mean (sample size)	138.80 (n = 56)	139.04 (n = 55)	136.37 (n = 60)
Cl (mmol/L)	Between Lot	0.56	0.00 *	0.00 *
	Within Lot	0.94	0.60	0.73
	Total	1.09	0.60	0.73
	Mean (sample size)	104.21 (n = 56)	105.00 (n = 55)	103.38 (n = 60)
Ca (mg/dL)	Between Lot	0.24	0.00 *	0.37
	Within Lot	0.94	0.85	0.91
	Total	0.97	0.85	0.98
	Mean (sample size)	8.37 (n = 53)	8.58 (n = 51)	8.70 (n = 62)

Table S18. Total percent (%) CV and components of variance between lots and within lots based on site and analyte for liver function tests.

		**Mean % CV**		
**Analyte (Unit)**	**Variance Component**	**Site 1**	**Site 2**	**Site 3**
AST (U/L)	Between Lot	3.94	2.12	1.03
	Within Lot	4.77	9.00	2.97
	Total	6.19	9.25	3.15

	Mean (sample size)	17.75 (n = 56)	20.05 (n = 55)	21.87 (n = 62)
ALT (U/L	Between Lot	1.41	0.00 *	1.94
	Within Lot	6.35	5.30	7.49
	Total	6.50	5.30	7.73
	Mean (sample size)	11.59 (n = 56)	12.49 (n = 55)	16.20 (n = 61)
ALP (U/L)	Between Lot	0.37	0.00 *	0.75
	Within Lot	1.64	2.09	1.50
	Total	1.69	2.09	1.68
	Mean (sample size)	86.34 (n = 56)	70.93 (n = 56)	68.81 (n = 62)
Total Bilirubin (mg/dL)	Between Lot	2.10	0.00 *	0.22
	Within Lot	4.71	3.68	3.92
	Total	5.16	3.68	3.93
	Mean (sample size)	0.30 (n = 53)	0.35 (n = 50)	0.31 (n = 60)
Albumin (mg/dL)	Between Lot	0.61	0.38	1.11
	Within Lot	2.34	2.15	1.81
	Total	2.42	2.19	2.12
	Mean (sample size)	4.01 (n = 56)	3.88 (n = 52)	4.16 (n = 62)
Total Protein (mg/dL)	Between Lot	0.81	0.00 *	0.81
	Within Lot	1.80	1.14	1.42
	Total	1.97	1.14	1.63
	Mean (n)	6.74 (n = 56)	6.50 (n = 51)	6.75 (n = 62)

Table S19. Total percent (%) CV and components of variance between lots and within lots based

on site and analyte for kidney function tests.

Analyte (Unit)	Variance Component	Mean % CV		
		Site 1	Site 2	Site 3
BUN (mg/dL)	Between Lot	0.00 *	0.13	0.00 *
	Within Lot	2.94	2.04	2.60
	Total	2.94	2.04	2.60
	Mean (sample size)	11.56 (n = 55)	17.04 (n = 51)	13.01 (n = 62)
Creatinine (mg/dL)	Between Lot	0.17	0.00 *	0.79
	Within Lot	2.79	2.64	2.76
	Total	2.79	2.64	2.87
	Mean (sample size)	0.82 (n = 55)	0.83 (n = 50)	0.69 (n = 60)

Table S20. Total percent (%) CV and components of variance between lots and within lots based on site and analyte for lipid tests.

Analyte (Unit)	Variance Component	Mean % CV		
		Site 1	Site 2	Site 3
Cholesterol (mg/dL)	Between Lot	0.46	0.10	0.79
	Within Lot	2.03	1.85	1.59
	Total	2.98	1.85	1.77
	Mean (sample size)	151.80 (n = 56)	149.83 (n = 52)	185.19 (n = 62)
Triglycerides (mg/dL)	Between Lot	0.00 *	0.00 *	0.00 *
	Within Lot	1.44	1.83	2.77
	Total	1.44	1.83	2.77

	Mean (sample size)	137.80 (n = 56)	108.92 (n = 52)	141.05 (n = 62)

Table S21. Total percent (%) CV and components of variance between operators and within operators based on site and analyte for electrolytes.

Analyte (Unit)	Variance Component	Mean % CV		
		**Site 1**	**Site 2**	**Site 3**
K (mmol/L)	Between Operator	1.48	0.00 *	0.39
	Within Operator	4.14	2.08	1.48
	Total	4.39	2.08	1.54
	Mean (sample size)	4.38 (n = 60)	4.70 (n = 60)	4.50 (n = 63)
Na (mmol/L)	Between Operator	0.00 *	0.17	0.00 *
	Within Operator	0.66	0.75	0.59
	Total	0.66	0.77	0.59
	Mean (sample size)	137.87 (n = 60)	139.28 (n = 60)	136.73 (n = 63)
Cl (mmol/L)	Between Operator	0.06	0.21	0.00 *
	Within Operator	0.82	0.83	0.97
	Total	0.82	0.86	0.97
	Mean (sample size)	103.15 (n = 60)	104.97 (n = 60)	103.95 (n = 63)
Ca (mg/dL)	Between Operator	0.17	0.00 *	0.23
	Within Operator	0.94	2.70	1.01
	Total	0.95	2.70	1.03
	Mean (sample size)	8.31 (n = 56)	8.52 (n = 51)	8.64 (n = 65)

Table S22. Total percent (%) CV and components of variance between operators and within operators based on site and analyte for liver function tests.

Analyte (Unit)	Variance Component	Mean % CV		
		Site 1	Site 2	Site 3
AST (U/L)	Between Operator	2.27	1.57	1.81
	Within Operator	7.29	8.16	2.72
	Total	7.63	8.31	3.26
	Mean (sample size)	18.02 (n = 60)	20.05 (n = 60)	21.61 (n = 66)
ALT (U/L)	Between Operator	2.74	0.00 *	0.00 *
	Within Operator	5.48	4.21	7.27
	Total	6.13	4.21	7.27
	Mean (sample size)	11.82 (n = 60)	12.73 (n = 60)	15.50 (n = 64)
ALP (U/L)	Between Operator	0.82	0.15	0.94
	Within Operator	1.50	1.24	1.65
	Total	1.71	1.25	1.90
	Mean (sample size)	86.22 (n = 60)	72.13 (n = 60)	68.14 (n = 66)
Total Bilirubin (mg/dL)	Between Operator	0.75	0.00 *	0.76
	Within Operator	5.20	4.09	3.14
	Total	5.25	4.09	3.23
	Mean (sample size)	0.29 (n = 57)	0.35 (n = 51)	0.31 (n = 62)
Albumin (mg/dL)	Between Operator	0.73	0.42	0.91
	Within Operator	2.13	1.68	1.78
	Total	2.25	1.73	2.00

	Mean (sample size)	4.00 (n = 60)	3.89 (n = 54)	4.11 (n = 66)
Total Protein (g/dL)	Between Operator	1.08	0.31	0.77
	Within Operator	1.71	1.22	1.42
	Total	2.02	1.26	1.62
	Mean (sample size)	6.78 (n = 60)	6.51 (n = 54)	6.70 (n = 65)

Table S23. Total percent (%) CV and components of variance between operators and within operators based on site and analyte for kidney function tests.

		**Mean % CV**		
**Analyte (Unit)**	**Variance Component**	**Site 1**	**Site 2**	**Site 3**
BUN (mg/dL)	Between Operator	0.00 *	0.34	0.40
	Within Operator	2.35	1.58	2.72
	Total	2.35	1.61	2.75
	Mean (sample size)	11.85 (n = 60)	16.96 (n = 54)	13.15 (n = 65)
Creatinine (mg/dL)	Between Operator	0.00 *	0.00 *	0.41
	Within Operator	3.16	2.47	2.72
	Total	3.16	2.47	2.75
	Mean (sample size)	0.81 (n = 59)	0.83 (n = 54)	0.68 (n = 62)

Table S24. Total percent (%) CV and components of variance between operators and within operators based on site and analyte for lipid tests.

		**Mean % CV**		
**Analyte (Unit)**	**Variance Component**	**Site 1**	**Site 2**	**Site 3**

Cholesterol (mg/dL)	Between Operator	0.93	0.00 *	0.97
	Within Operator	1.89	1.25	1.57
	Total	2.11	1.25	1.85
	Mean (sample size)	152.33 (n = 60)	149.17 (n = 54)	185.36 (n = 66)
Triglycerides (mg/dL)	Between Operator	0.36	0.00 *	0.12
	Within Operator	1.30	1.57	2.58
	Total	1.35	1.57	2.58
	Mean (sample size)	137.28 (n = 60)	109.17 (n = 54)	137.91 (n = 66)

Sample Contriving Method Validation Study

In the performance comparison study and precision study, select samples were modified in order to achieve extreme values for analytes' concentration. Briefly, blood samples from a single subject are collected into 10mL lithium heparin BCTs (Blood Collection Tubes) and transferred to a sterile 50mL conical tube. The blood samples are slightly diluted to modify the analyte concentration, and then drawn into lithium heparin ZDiscs or PSTs by butterfly tubing, similar to conventional blood draw as shown in the Supplementary Figure S1.

In order to validate the sample contriving method, analytes' concentrations in the double-heparinized blood samples, which were drawn from the test apparatus as shown in Figure S1, were compared to directly drawn whole blood for both ZDiscs and PSTs.

Blood samples were collected from 10 subjects. Each subject had blood directly drawn into one ZDisc as well as one PST (i.e. direct draw group) and two 10 mL Li-Heparin PSTs. The blood in Li-Heparin tubes were subject to the contriving method: blood samples were drawn into one ZDisc

and one PST (i.e. contrived group). No dilution was performed here in order to compare analyte concentration in the double-heparinized group (i.e. contrived group) vs the direct draw group. Blood samples in ZDiscs were separated using the ZDrive while samples in the PSTs were separated using a conventional centrifuge and tested for the concentration of analytes. Analyte concentration in plasma from both ZDisc and PST were measured using a chemistry analyzer, Piccolo Xpress Chemistry Analyzer (Abaxis, Union City, CA, USA). Note that 12 analytes excluding lipid analytes (i.e. cholesterol and triglycerides) were assessed in this study due to the limitation in the panel configuration of the chemistry analyzer.

Table S25 shows the grand mean and SD of analyte concentration across ZDiscs and PSTs, and mean % differences between the direct draw vs the contrived method for both ZDisc and PST. Notably, several analytes (Creatinine, ALT) showed the mean % difference close to or greater than 5%. Mean % difference between the direct draw vs the contrived method for creatinine was -12.37% for ZDisc and 9.76% for PST, however the paired t-test showed that the difference is not significant (p-value: 0.14 for ZDisc and 0.38 for PST). Similarly, mean % difference between the direct draw vs the contrived method for ALT is 4.39% and 8.99% for ZDisc and PST, respectively, but no significance determined in the paired t-test (p-value: 0.87 for ZDisc and 0.11 for PST). All other analytes showed less than 5% in the mean % difference for the direct draw and the contrived method.

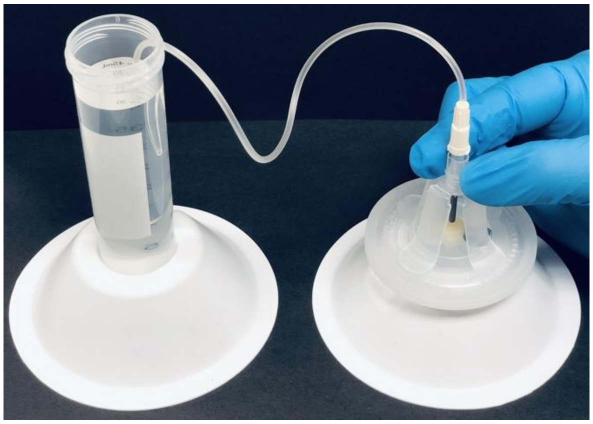

Figure S1. Modified blood collection set including 21G butterfly 12-inch tubing to transfer the modified samples in a 50mL conical tube into a ZDisc.

Table S25. Grand mean with SD and mean % difference between the direct draw group vs the contrived group for each analyte

		K	Na	Cl	Ca	BUN	Cre
**Total Mean (± SD)**		4.08 ± 0.26	141.48 ± 1.99	103.35 ± 1.96	2.39 ± 0.09	5.20 ± 1.37	78.80 ± 18.25
**Mean condition analyte concentration (± SD)**	**ZDisc (Contrived)**	4.1±0.2	141.4±2.0	103.2±1.8	2.4±0.1	5.2±1.4	72.5±20.9
	**ZDisc (Direct draw)**	4.2±0.4	142.1±2.1	103.4±2.3	2.4±0.1	5.2±1.4	84.4±16.9
	**PST (Contrived)**	4.0±0.2	141.2±2.3	103.6±2.0	2.4±0.1	5.2±1.4	81.8±18.3
	**PST (Direct draw)**	4.0±0.2	141.2±1.5	103.2±1.9	2.4±0.1	5.2±1.4	76.5±17.1
**Mean % difference**	**ZDisc**	-3.08	-0.49	-0.17	1.09	-0.02	-12.37
	**PST**	-0.37	0.00	0.40	0.74	0.95	9.76
		**AST**	**ALT**	**ALP**	**T.Bili**	**Alb**	**TP**
**Total Mean (± SD)**		30.38 ± 6.13	33.13 ± 15.20	62.48 ± 7.47	17.35 ± 3.75	41.20 ± 3.34	75.00 ± 7.05
**Mean condition analyte concentration (± SD)**	**ZDisc (Contrived)**	31±6.6	33.1±15.5	63.4±7.0	17.2±3.9	41.7±3.1	75.3±7.4
	**ZDisc (Direct draw)**	30.2±6.6	32.9±17.0	61.9±8.9	17.4±3.8	40.5±3.7	75.4±7.9

	**PST (Contrived)**	30.7±5.9	34.4±15.5	62.2±6.8	17.5±4.0	42.1±3.3	75.1±6.4
	**PST (Direct draw)**	29.6±6.3	32.1±15.1	62.4±8.1	17.3±3.9	40.5±3.3	74.2±7.5
**Mean % difference**	**ZDisc**	3.05	4.39	1.75	-1.21	3.15	0.00
	**PST**	4.25	8.99	0.91	1.04	4.04	1.40

Lowest Quartile Performance Comparison Analysis

In order to assess whether use of the ZDisc might impact analytical limit of detection, as secondary analysis the bottom quartile of reference sample results was analyzed separately. Performance comparison fit and bias for this sub-set were generally comparable to those found for analysis of all samples for each of the 14 analytes.

Table S26. Mean percent bias for all analytes at each site and for each chemistry analyzer for the lowest quartile subgroup

Site/Analyzer		K	Na	Cl	Ca	AST	ALT	ALP
**Site 1**	**Analyzer 1**	1.279	0.503	0.057	0.133	-0.730	3.590	2.482
	**Analyzer 2**	0.042	-0.356	-0.090	-0.268	-0.338	1.873	-0.410
	**Analyzer Pooled**	0.661	0.073	-0.017	-0.067	-0.534	2.726	1.036
**Site 2**	**Analyzer 1**	0.043	-0.275	-0.472	0.436	-0.110	0.071	1.209
	**Analyzer 2**	-0.648	0.265	0.498	0.559	-2.765	1.382	1.537
	**Analyzer Pooled**	-0.685	-0.005	0.431	-0.144	-4.124	1.037	3.032
**Site 3**	**Analyzer 1**	0.066	0.094	0.133	0.178	-0.449	-0.201	-0.202
	**Analyzer 2**	-1.934	0.181	0.461	0.559	-2.192	-5.221	0.076
	**Analyzer Pooled**	-2.672	0.137	0.365	-0.144	-3.125	-2.034	0.366
**3 sites pooled / Analyzer 1**		-1.193	0.109	0.198	0.603	-2.848	1.618	2.392
**3 sites pooled / Analyzer 2**		-0.930	0.015	0.226	-0.107	-1.471	-1.387	0.420
**Total**		-1.062	0.062	0.212	0.248	-2.159	0.116	1.406
**Site/Analyzer**		**Total Bilirubin**	**Albumin**	**Total Protein**	**BUN**	**Creatinine**	**Cholesterol**	**Triglycerides**
**Analyzer 1**		0.000	1.559	0.899	1.111	0.077	N/A	N/A
**Analyzer 2**		7.043	1.329	1.318	0.256	2.161	-0.938	-0.026
**Analyzer Pooled**		3.522	1.444	1.109	0.683	1.119	-0.938	-0.026
**Analyzer 1**		0.000	3.428	0.913	-2.872	-0.255	N/A	N/A
**Analyzer 2**		-0.170	1.717	-0.529	0.000	2.534	0.221	-0.675
**Analyzer Pooled**		-0.085	2.573	0.192	-1.436	1.140	0.221	-0.675
**Analyzer 1**		0.000	0.412	0.606	0.386	-0.282	N/A	N/A
**Analyzer 2**		-3.756	1.969	0.863	0.980	2.277	-0.709	0.883
**Analyzer Pooled**		-1.878	1.190	0.735	0.683	0.997	-0.709	0.883

**3 sites pooled / Analyzer 1**	0.000	2.143	0.862	-0.316	-0.102	N/A	N/A
**3 sites pooled / Analyzer 2**	0.401	1.610	0.298	0.436	2.279	-0.412	0.131
**Total**	0.200	1.877	0.580	0.060	1.088	-0.412	0.131

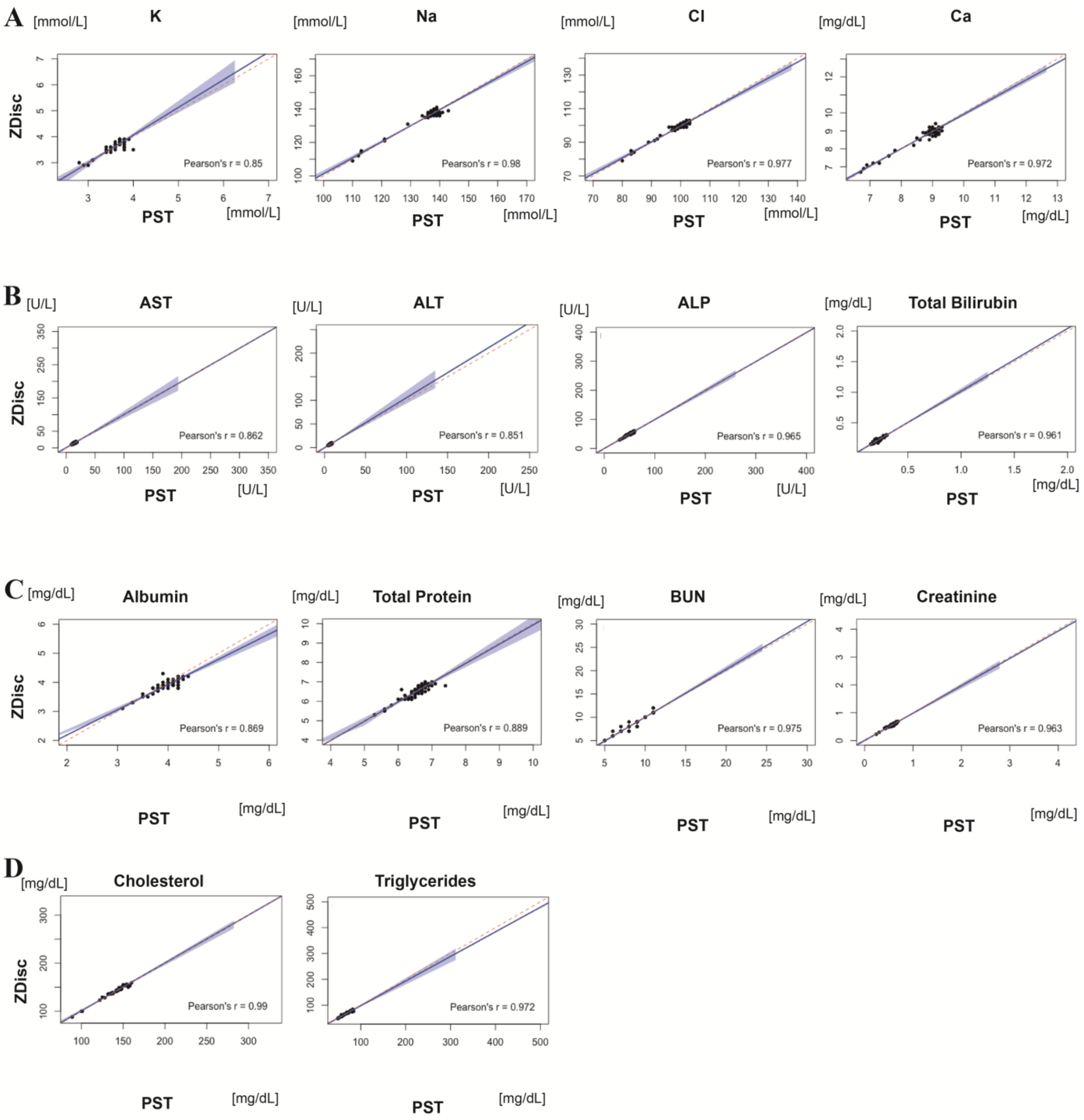

Figure S2. Scatter plots of the lowest quartile subgroup representing all 14 analytes results for ZDisc vs. vacutainer PST with Deming regression line with 95% CI (blue), reference interval for each analyte (orange), and identity line (red dots) across all sites and analyzers (A thru D); Bland-Altman plots with mean (red solid line) and +/- 2*SD (red dotted line) (E thru H).

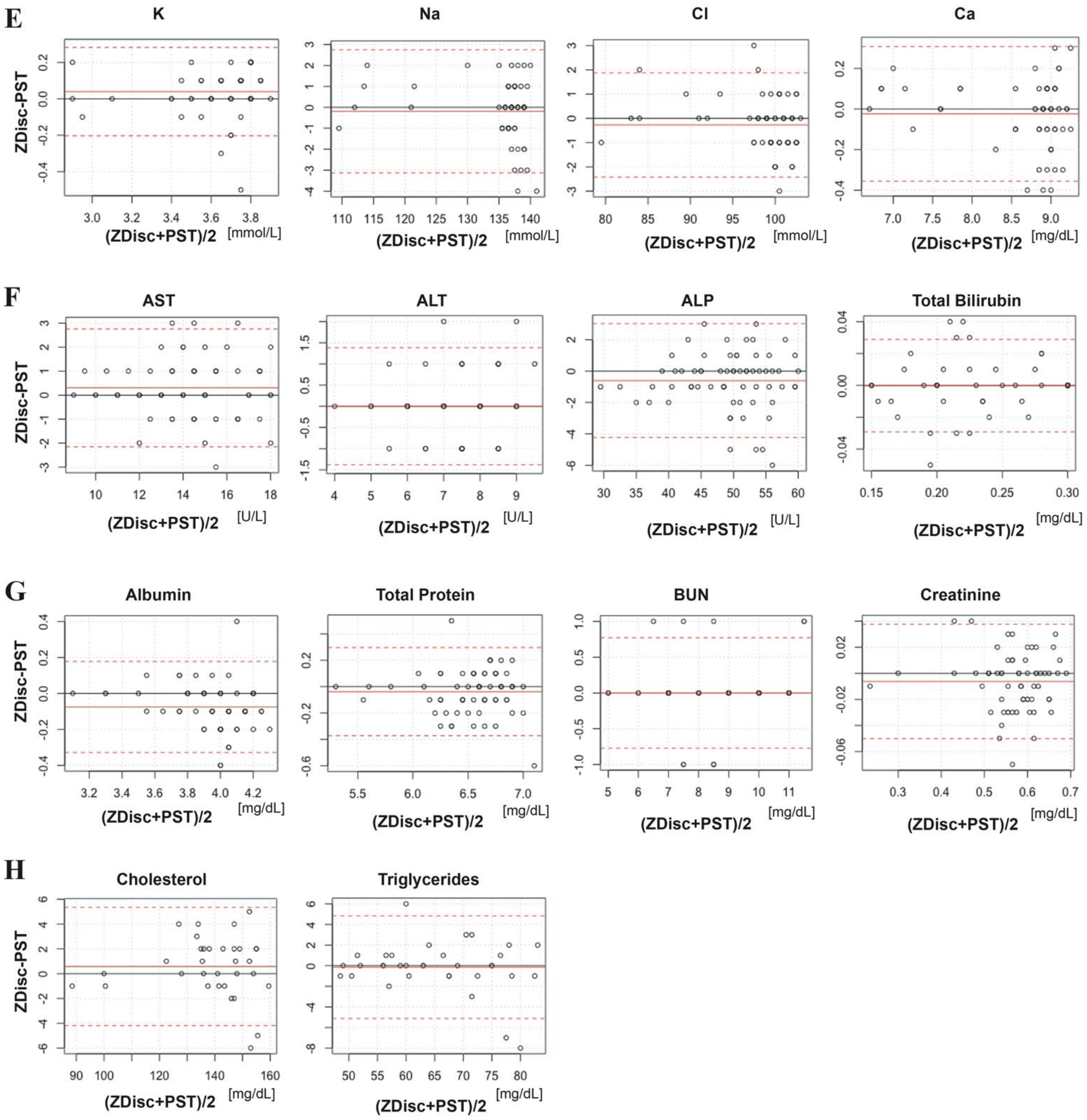

Figure S2. (continued) Scatter plots of the lowest quartile subgroup representing all 14 analytes results for ZDisc vs. vacutainer PST with Deming regression line with 95% CI (blue), reference interval for each analyte (orange), and identity line (red dots) across all sites and analyzers (A thru D); Bland-Altman plots with mean (red solid line) and +/- 2*SD (red dotted line) (E thru H).
